# Clinicogenomic Insights for Progression-Free Survival in Prostate Cancer

**DOI:** 10.3390/ijerph23020256

**Published:** 2026-02-18

**Authors:** Kelvin Ofori-Minta, Bofei Wang, Jonathon E. Mohl, Abhijit Mandal, Ming-Ying Leung

**Affiliations:** 1 Department of Mathematical Sciences, The University of Texas at El Paso, El Paso, TX 79968, USA; koforimint@miners.utep.edu (K.O.-M.); jemohl@utep.edu (J.E.M.);; 2 Department of Leukemia, Division of Cancer Medicine, The University of Texas MD Anderson Cancer Center, Houston, TX 77030, USA; bwang8@mdanderson.org; 3 Border Biomedical Research Center, The University of Texas at El Paso, El Paso, TX 79968, USA

**Keywords:** oncology, prostate cancer, clinical data, single nucleotide variants, progression free survival modeling, penalized cox model, machine learning, Harrell’s concordance index

## Abstract

**Highlights:**

**Public health relevance—How does this work relate to a public health issue?**

- Prostate cancer remains one of the most prevalent malignancies among men worldwide, with substantial morbidity, mortality, and healthcare burden, driven by heterogeneous disease progression patterns.
- This study leverages survival analysis models to identify patient profiles at elevated risk of prostate cancer progression, aiming to generate hypotheses for investigation that can support more targeted clinical intervention and patient monitoring.

**Public health significance—Why is this work of significance to public health?**

- This work revealed insights into prostate cancer by showing that key clinical factors remain primary drivers of progression risks, while genomic factors provide additional information on disease progression and potential biological mechanisms.
- This combined clinicogenomics perspective supports biologically informed risk stratification and facilitates hypothesis generation.

**Public health implications—What are the key implications or messages for practitioners, policy makers, and/or researchers in public health?**

- Progression-free survival models can be used to help distinguish patients at higher risk of cancer progression from those with more stable conditions, potentially reducing overtreatment and focusing resources where they are most needed.
- The findings highlight the value of integrating genomic and clinical data for prostate cancer evaluation and monitoring, which offers a foundation for assessing progression risks, thereby enabling biologically informed cohort-level disease risk assessment and stratification.

**Abstract:**

Prostate cancer (PrCa), the second most common cancer diagnosed in men globally, remains a critical challenge in precision oncology. While PrCa can be deadly, it is highly treatable if detected early. Identifying associative factors influencing disease progression risks can help inform preliminary steps that will further the expedition of clinical therapeutic intervention decisions, which will improve treatment outcomes. While conventional PrCa progression assessment tools rely heavily on a few clinical parameters, the importance of genomic information is increasingly recognized. In this study, we evaluate the prognostic value of patients’ clinicogenomic profiles in modeling progression-free survival (PFS) of PrCa. Three survival models, namely the penalized Cox model, random survival forest, and a deep learning survival neural network, were deployed with extensive tuning applied to a dataset for a cohort of 494 patients with PrCa. This dataset, compiled from public data in The Cancer Genome Atlas (TCGA) accessed via cBioPortal, consists of relevant clinical features and single-nucleotide variant information on likely PrCa-related genes. The survival models demonstrated satisfactory discriminatory performance, with Harrell’s concordance index ranging from approximately 0.80 to 0.87 on held-out test data, indicating their ability to rank patients according to their relative progression risk among patients, while exhibiting distinct dynamics, all three models consistently identified clinical variables that indicated neoadjuvant treatment history, neoplasm cancer status, and tumor recurrence as well as the gene *MYH6* as important predictor variables for PrCa PFS. Our findings suggest the incorporation of genomic data into the survival modeling workflow, thereby allowing the use of integrated clinicogenomics information to gain insights into progression risks for patients with PrCa.

## 1. Introduction

Prostate cancer (PrCa) is a tumor formed when cells grow and multiply abnormally in and around the prostate gland. When metastasized (migrated to other parts of the body), it can lead to terminal and aggressive forms with poor diagnosis [1,2]. Globally, PrCa is the second-most common cancer diagnosed in men, and for more than half of the global population, it is the most endemic cancer in men and the leading cause of death in Central America and Sub-Saharan Africa [3,4]. The American Cancer Society Cancer Facts and Figures (2025) show an estimated 35,770 deaths from 313,780 new cases of PrCa [3]. The prostate-specific antigen test is commonly used for screening PrCa, and more recently, some blood-based and urine-based biomarkers like PTEN and PCA3 have been reported to be promising diagnostic tools [5,6].

While PrCa can be deadly, it is highly treatable if detected early. However, the five-year survival rate declines significantly from close to 100% for early stages to less than 40% when the cancer has progressed to advanced metastatic stages [3], underscoring the importance of uncovering and understanding the clinical and biological factors influencing PrCa progression. The clinical course of PrCa varies widely, while some patients exhibit dormant forms where cancer cells persist without significant growth for years, others experience aggressive metastasis despite early treatment [7,8,9].

A clinically relevant endpoint in many cancer studies is the progression-free survival (PFS), which refers to the time from initiation of treatment until disease progression or death, whichever comes first [10]. PFS serves as an indicator of cancer stabilization and has been earmarked as a proxy endpoint for overall survival (OS) that measures the time from treatment initiation until death. Compared to OS, PFS can be measured sooner with less cost, it provides earlier insight into treatment effect, and also quickens the drug development process and approval [11]. Thus, accurate risk stratification models for PFS are crucial for tailoring intervention intensity, monitoring clinical procedures, and informing patient counseling.

Recently, PFS predictions for PrCa based on certain clinical factors have been discussed (see [10]). At the same time, high-throughput sequencing technologies have been made available via large-scale genomic sequencing data, opening the opportunity to integrate genomic and clinical profiles of patients, giving rise to the field of clinicogenomics that analyzes clinical and genomic features together for improved prognostic and predictive modeling. As reviewed in [12], many studies have successfully utilized genomics profiles to identify PrCa-associated genes. Recent works [13,14,15] provided further examples demonstrating the usefulness of genomic information to shed light on various biomedical aspects of PrCa. Combined clinical and omics data from the TCGA PrCa dataset have been used to construct prognostic models to help predict biochemical recurrence and postsurgical PFS [16,17]. However, the use of clinical and genomic single-nucleotide variant (SNV) information together for modeling PrCa PFS has not yet been reported to date.

In this paper, we present our exploration into possible associative predictors of PrCa PFS using multiple survival modeling frameworks applied to clinical and genomic information for a cohort of 494 patients with PrCa. Our objective is to identify key clinical and genomics factors associated with PrCa PFS that can be used to generate hypotheses for further in-depth investigation. The clinical data contains information on patients’ demographics, treatment history, disease status, and survival times. The genomics data comprises SNVs extracted from the corresponding patients’ whole exome sequencing results. The SNV information allowed us to identify 27 likely PrCa-related (LPC) protein-coding genes based on the variants’ occurrence frequencies and functional effects, as well as downstream bioinformatics analyses. Combining such clinical and genomics information, we compiled a clinicogenomics dataset for this cohort. The survival modeling methods used to predict PrCa PFS include a traditional penalized Cox model (PCM), a well-established random survival forest (RSF) method, and a deep learning survival model (DeepSurv). While PCM captures linear relationships between variables and also provides interpretable estimates of covariate effects, RSF and DeepSurv capture non-linear, latent relationships and also rank covariates based on their relative contributions to PFS.

In this study, we aim to harness these modeling strategies to offer insights into PrCa progression by systematically analyzing the patients’ PFS using the combined clinicogenomics dataset to uncover factors associated with PFS. We also compared the effectiveness of the three statistical and machine learning models in PrCa PFS prediction. It is anticipated that clinicogenomics data modeling with multiple approaches will help reveal the most important covariates associated with PrCa PFS, which may not be apparent when clinical data is analyzed alone. As such, understanding how specific genomic alterations, treatment exposures, and clinical features interact to influence PrCa PFS is a crucial preliminary step in advancing personalized treatment guidelines for oncology. Ultimately, this integrative clinicogenomics approach has the potential to refine PFS risk stratification modeling to inform more precise and personalized therapeutic interventions.

## 2. Materials and Methods

**Figure 1:**
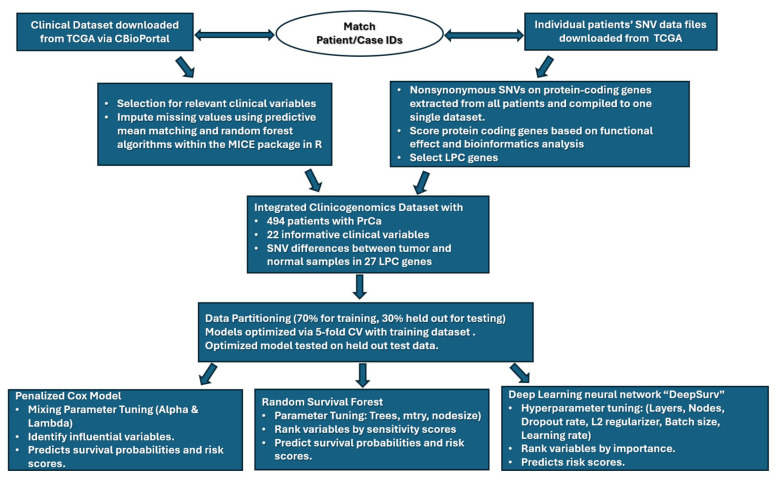
Main steps of clinical and genomics data collection, integration, and PFS analyses with three survival analysis models. Clinical and genomic SNV data for prostate cancer patients were obtained from TCGA and matched by case identifiers. Key clinical variables were selected, and missing values were imputed using MICE, while non-synonymous SNVs were functionally scored to select LPC genes. An integrated dataset of 494 patients (22 clinical variables and 27 LPC genes) was partitioned into training (70%) and testing (30%) sets, with 5-fold cross-validation for model optimization. Three survival models were trained to estimate survival probabilities, relative risk and relative variable importance based on model-specific procedures.

The methodological procedure developed for this work is summarized in Figure 1. This flowchart shows the main steps of data collection, integration and analysis. Clinicogenomics features were constructed by integrating curated clinical variables with selected SNV-based genomic features. Model development followed a consistent workflow across all modelling approaches, including data preprocessing, partitioning and parameter tuning strategies, performance assessment methods. Extended tuning, diagnostic plots and all workflow implementation details are available as Supplementary Materials (SF01_pipeline_modules_data and SF02_data_processing) and are also publicly accessible through the project’s GitHub repository—https://github.com/kelvin-meyet/ClinicoGenomicInsights (accessed on 5 February 2026).

### 2.1. Clinical Data Preparation

Clinical data source for this work is the cBioPortal for cancer genomics, an interactive open-source platform that has made cancer omics profiles accessible to researchers and physicians [18,19]. Using their representative state transfer application programming interface (REST-API) and localized storage, clinical data in The Cancer Genome Atlas Prostate Adenocarcinoma (TCGA-PRAD) project was accessible for this study. We initialized, queried, and extracted data that encompassed 55 clinical features from 494 patients with the API client via R software version 4.4.1. From these, 22 clinically relevant and informative features (see Table 1), excluding identifier columns, were selected for downstream analyses with PFS Status and PFS Months as target variables and the remaining 20 features as clinical predictors. Description of clinical variables can be found in Supplementary Materials (SF03_clinical_variables).

Several clinical covariates contained missing values and were imputed to enable the construction of a complete clinical feature matrix (see Table 1). Missing data were addressed using Multivariate Imputation by Chained Equations (MICE) with Predictive Mean-Matching (PMM) and Random Forest (RF) methods [20]. Imputation models were fitted on the full clinical dataset prior to model development to obtain a complete set of clinical data that can be harmonized with genomic features. PFS months and event status were included as helping variables in the imputation model to preserve associations among observed variables; however, imputed outcomes were not used as prediction targets. A single completed dataset was obtained from the imputation cycles for downstream modeling. Distributional comparisons and visual diagnostics were used to assess the plausibility of imputed values. Subsequent data partitioning, model training, hyperparameter tuning, and evaluation were performed strictly after imputation, with the held-out test set excluded from all model-fitting procedures. The complete set of plots showing the imputation results for all imputed features before and after imputation is in the Supplementary Materials (SF04_imputation). Thus, results are interpreted as internally validated and exploratory rather than inferential.

### 2.2. Genomics Data Preparation

We downloaded 503 variant call format (VCF) files containing genomic SNV information from TCGA. Each of these files corresponded to only one individual patient, but we found five pairs of files with the same patient ID. The SNV information for each of these duplicate pairs was combined, resulting in 498 files with genomics data. However, only 494 of these have patient identifiers matching those in our clinical dataset. Each VCF file contained SNV information from a patient’s tumor and normal samples. This procedure is described in [21], where these data were analyzed, and the nonsynonymous SNVs (i.e., those that can cause changes in the encoded amino acids) on protein-coding regions were identified. A scoring function based on two popular functional effect analysis tools, FATHMM [22] and PROVEAN [23] were then employed to calculate the cumulative deleterious effects of these SNVs on protein-coding genes as follows.

For any protein-coding gene g, we calculated its cumulative pathogenicity score P(g) by:

$$P\left(g\right)=\frac{1}{\ln L\left(g\right)}\sum_{v\;\mathrm{in}\;g}\mathrm{RankScore}\left(v\right)\times [s\left(v,\;\mathrm{tumor}\right)-s\left(v,\;\mathrm{normal}\right)]$$

where L(g) is the total coding sequence length for gene g, $\mathrm{RankScore}\left(v\right)$ is the average rank of the deleterious functional effects of the variant v as assessed by FATHMM and PROVEAN, and s(v, tumor) and s(v, normal) are the numbers of subjects with the pathogenic variant v in the tumor and normal samples.

Subsequent bioinformatics analysis was conducted on the top 1% genes with the highest $P\left(g\right)$ scores. Analysis of the protein–protein interaction of these top genes with a compiled list of known PrCa-related genes (based on published literature and databases), and selecting those genes with above average interactions led to 27 likely PrCa-associated (LPC) genes: *NKX3.1*, *CSMD3*, *TRRAP*, *CHD4*, *VWF*, *EPHB1*, *HERC2*, *MCM3*, *SPTA1*, *SALL1*, *HERC1*, *RYBP*, *TTN*, *CHD5*, *MYH6*, *FAT3*, *ATM*, *KMT2D*, *FOXA*, *TP53*, *SPOP*, *SMAD4*, *LRP1B*, *IDH1*, *CTNNB1*, *BRAF*, and *KMT2C* [24]. The SNV data for each individual patient in the cohort were examined to obtain the counts of deleterious SNVs on these 27 genes. Then, the difference between the deleterious SNV counts in each gene between the tumor tissue and the normal tissue of the patient was used as a genomic feature. Essentially, the 27 selected genomic features reflected the cumulative deleterious effects of the SNVs on these genes.

### 2.3. The Clinicogenomics Dataset

The merging of unique patient clinical identifiers (Patient ID) with their corresponding genomic case identifiers (Case ID) produced an integrated dataframe of clinical and genomic data from 494 patient rows and 49 columns (22 clinical plus 27 genomic features). Again, the codes used for this task can be found in Supplementary Materials (SF02_data_processing).

In this study, the dataset was partitioned into training and testing subsets (70:30) using the sample.split function from the CaTools package in R, ensuring proportional representation of survival outcome (PFS) status across both sets prior to training. For the DeepSurv model, cross-validation folds within the training set were explicitly stratified by PFS status to mitigate event imbalance. For PCM and RSF, internal resampling procedures inherent to each method were used, which accommodated right-censored data but did not explicitly enforce stratified cross-validation folds. The training set (70%) was used for model training and hyperparameter tuning via cross-validation, while the held-out test set (30%) was reserved exclusively for model evaluation. All predictors and outcome variables were preprocessed for compatibility with survival modeling techniques. Distribution of PFS time and status was first explored separately within the training and test sets to ensure consistency of PFS objects. This was aimed at assessing the class imbalance of our survival endpoint. We also explored PFS status for all patients during the study period, as well as the follow-up times within the censored and non-censored groups. Summary statistics of PFS time were computed separately for censored and non-censored groups, which enabled the detection of early or late censoring patterns within the follow-up times. Univariate Kaplan–Meier (KM) survival analysis was conducted to primarily investigate the influence of selected covariates on PFS [25]. Specific covariates of interest, such as radiation therapy (RT), history of neoadjuvant treatment (HNT), neoplasm cancer status (NCS), and new tumor after initial treatment (NTAIT), were individually stratified into categorical levels. KM survival curves were estimated for each covariate, and the log-rank test was used to assess the statistical significance of survival difference across each stratum [26]. See exploratory_analysis.R file in Supplementary Materials (SF01_pipeline_modules_data).

### 2.4. Survival Modeling Methods

The three survival modeling approaches employed in this study were selected to reflect complementary methodological strengths. The PCM was included for its interpretability and ability to perform variable selection in the presence of correlated predictors. The RSF was chosen to capture non-linear relationships and higher-order interactions without requiring parametric assumptions. DeepSurv was included as a complementary deep-learning-based extension of the Cox model to explore complex, potentially high-dimensional clinicogenomics interactions. These models provide a synergistic balance between interpretability, flexibility, and expressive capacity.

All multivariate survival models used in this study are inherently designed to accommodate right-censored survival data. PCM and DeepSurv optimize the partial likelihood-based objective function, which directly incorporates censoring, while RSF employs log-rank-based splitting rules that also incorporate censored observations during tree construction. No additional re-weighting was applied to censored events, as our primary objective was comparative risk stratification in this single cohort rather than unbiased estimation of precise PrCa PFS risk prediction. As such, our models employed are censoring-aware and not censoring-adjusted. We applied these models to estimate the PFS within the integrated clinicogenomics dataset, with PFS months (time to PrCa progression) and PFS status (disease progression observed or not) as target survival objects for modeling PrCa PFS risk stratification rather than as the precise surrogate for overall survival per definition. Each modeling approach involved model-specific hyperparameters that were optimized within the training set using internal cross-validation and resampling procedures.

Model performance across all models was evaluated using Harrell’s concordance index (C-index) [27,28], which measured the ability of a model to correctly rank patients according to their relative risk of progression while accounting for right-censored observations through pairwise compatibility. The C-index assesses the discriminatory performance but does not evaluate calibration or accuracy of absolute risk estimates. Thus, results were interpreted in terms of relative risk stratification rather than precise probability prediction. Formal calibration analyses, such as time-dependent Brier scores or calibration curves [29], were not conducted but would be an important direction for future validation.

The C-index was computed separately for the training and held-out test sets and interpreted as a measure of discrimination rather than calibration or absolute risk accuracy. All models were implemented using survival-specific objective functions or splitting rules that inherently accounted for right-censored data. The PCM employed partial likelihood-based estimation with regularization, RSF utilized log-rank splitting rules with a bootstrapped aggregation framework, and DeepSurv optimized a Cox partial likelihood loss function using a neural network. Model implementations, hyperparameter optimization procedures, and performance evaluations are provided in the multivariate-analysis.R file in Supplementary Materials (SF01_pipeline_modules_data).

#### 2.4.1. PCM: A Penalized Survival Model

PCM was employed using the glmnet package in R [30] with elastic net penalization to balance variable selection and model shrinkage via a 5-fold cross-validation (CV) optimization and parameter tuning framework within the training dataset only, based on Harrell’s C-index. Predictors with non-zero coefficients under the optimal penalty were retained and subsequently used to refit a final standard Cox proportional hazards model to facilitate descriptive interpretation of relative associations and survival probabilities.

To assess the stability of model evaluation with respect to training data resampling, the model fitting and tuning procedure was repeated across multiple random refits within the training set while preserving the fixed held-out test set. In each iteration, optimal parameters were selected independently, and model discrimination was evaluated on the held-out test set. Reported mean and standard deviation values reflect between-run variability arising from internal resampling rather than uncertainty due to test set resampling. Model discrimination was assessed using Harrell’s C-Index on both training and test sets.

Because variable selection was performed prior to refitting the unpenalized Cox model, statistical inference from the refitted model should be interpreted cautiously; thus, standard errors and *p*-values do not account for uncertainty introduced by the selection process and may be optimistically biased. Accordingly, reported hazard ratios (HRs) are interpreted as measures of association rather than causal effects, with emphasis on directional and relative importance over precise effect estimation. Survival probabilities and relative risk estimates were derived using the survex package [31].

#### 2.4.2. RSF: A Tree-Based Machine Learning Method for Survival Analysis

The random survival forest (RSF) model was implemented using the randomForestSRC package [32,33] on the same processed training dataset. Model development employed bootstrapped aggregation with log-rank splitting rules to accommodate right-censored observations. Hyperparameters, including the number of trees (ntree), number of random variables randomly selected at each split (mtry) and minimum node size, were tuned using internal resampling procedures within the training set. Following hyperparameter selection, a final RSF model was trained on the full training dataset and evaluated on the held-out test. To assess the variability in model performance arising from resampling and tuning, the RSF fitting and evaluation procedure was repeated across multiple refits using different random seeds, while maintaining a fixed test set. Model discrimination was evaluated using Harrell’s C-Index on both training and test sets, with reported mean and standard deviation reflecting between-run variability. Feature importance was assessed using the VIMP metric from the minimal depth criterion [34,35]. Survival probabilities and relative risk estimates were obtained in the same way as the PCM.

#### 2.4.3. DeepSurv: A Deep Learning Neural Network Model for Survival Analysis

This model was included as a nonlinear comparator to explore the potential complex interactions between clinical and genomic features and PFS risk stratification. Model training and evaluation followed stratified training and held-out partitions. DeepSurv [36] model was implemented using Keras in R [37]. Numeric variables in the training set were scaled using the min-max normalization, and categorical variables were one-hot encoded. Transformation parameters learned from the training dataset were applied to t to the held-out test set. Model development and hyperparameter tuning were conducted exclusively within the training dataset. Hyperparameters (number of hidden layers, nodes, dropout rate, learning rate and L2 regularization) were tuned using 5-fold CV within the training set only. The optimal hyperparameter combination was selected based on the highest cross-validated Harrell’s C-index. Using the selected hyperparameters, a final DeepSurv model was trained on the full training dataset using the Scaled Exponential Linear Unit (SELU) activation function. Early stopping and adaptive learning rate adjustments were applied during training to mitigate overfitting, and the model was evaluated on a held-out test set [38,39,40,41].

For interpretability, a gradient-based variable importance approach was implemented to assess the sensitivity of the model’s predicted risk score to perturbations in individual input features [42,43]. Importance scores were computed as the mean absolute gradient across samples. DeepSurv provides the log-risk scores, which were exponentiated to obtain relative risk estimates, where higher risk scores imply higher hazards of PrCa progression. Harrell’s C-index is once again used to assess model discrimination. Given the stochastic nature of neural network optimization and the modest sample size with limited events, DeepSurv results are interpreted as complementary rather than confirmatory.

## 3. Results

We will first describe the compiled clinicogenomics dataset along with summary statistics and the univariate KM analysis results. Then, the PFS risk stratification insights based on PCM, RSF and DeepSurv will be presented.

### 3.1. Compiled Clinicogenomics Dataset and the PFS Distribution

The final compiled dataset (see prca_clinicogenomics_data in Supplementary Materials SF01_pipeline_modules_data) combined both clinical and genomics information of the 494 patients. Our target response is made up of a pair of variables. The first variable is the binary PFS status with 1 indicating an observed PrCa progression event and 0 otherwise. The second variable, “PFS months”, is a measure of the PFS time in months. The other columns in the dataset provide observed and imputed values of the 22 selected clinical covariates and the SNV frequency difference (tumor–normal) in the 27 selected LPC genes as described earlier.

**Figure 2:**
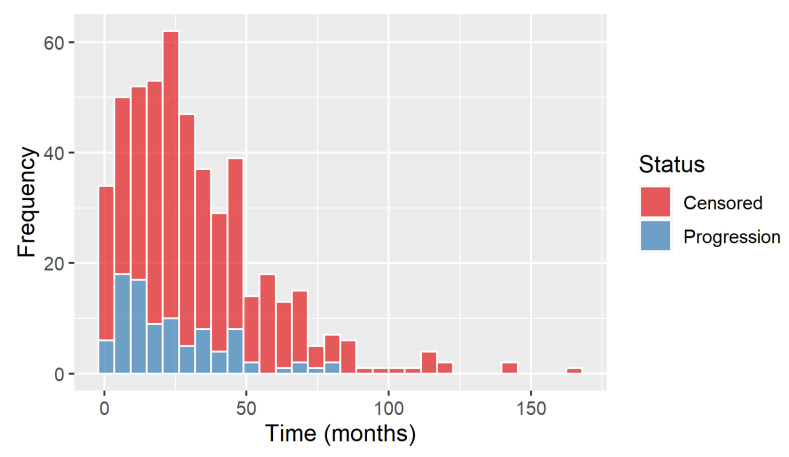
Distribution of progression-free survival status of all PrCa patients over time.

Figure 2 displays the distribution of PFS status during the study period; the higher proportion of censored events is an indicator that more patients were lost during follow-up or did not experience PrCa progression within the study period. The limited number of patients beyond 100 months suggests the scarcity of long-term data.

The summary statistics shown in Table 2 indicate a clear distinction between the two groups. We have 401 patients (81.2%) censored, while the remaining 93 patients (18.8%) experienced progression. For the censored group (subjects yet to experience progression), the follow-up periods tend to be longer as reflected by the higher means and medians and the wider ranges. In contrast, the observed progression group shows shorter follow-up times on average, suggestive of earlier occurrence of disease progression.

To ensure representativeness of the evaluation cohort, we examined the distribution of PFS times in both training and test sets. The distributions were found to be comparably similar since both training and test datasets displayed a right-skewed distribution of PFS time, where most events occur earlier in follow-up, and longer tails representing patients who remain progression-free for substantially longer periods (see Supplementary Materials SF05_kmcurves_and_plots, Figure S1).

### 3.2. KM Survival Analysis with Binary Clinical Predictors

**Figure 3:**
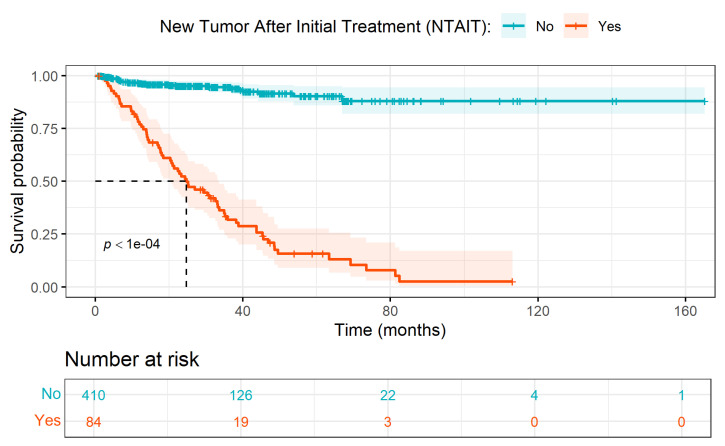
Kaplan–Meier (KM) survival curves showing progression-free survival (PFS) months, stratified by the presence of new tumor after initial treatment (NTAIT) (Yes vs. No). Shaded regions represent 95% confidence intervals, and tick marks indicate censored observations. The dashed line denotes the median PFS for the group experiencing NTAIT. The number-at-risk table is shown below the plot. Group differences were assessed using the log-rank test (*p* < 0.0001).

We estimated survival probabilities over time based on the binary clinical variables using KM survival curves. The few variables that made a significant difference for PrCa PFS with *p*-value < 0.05 are NTAIT, RT, NCS, and HNT. We present the KM curve for NTAIT in Figure 3, while Supplementary Materials SF05_kmcurves_and_plots (Figures S2 and S3) contain those for the other clinical variables.

The univariate effect of NTAIT on PFS shown in Figure 3 indicates that the non-persistent-tumor group maintains high chances of PFS throughout; the curve flattens early and remains near 0.9, indicating minimal progression events over time. For the persistent-tumor group, there is a steep decline in PFS, especially within the first 40 months, reflecting a high incidence of PrCa progression; the dotted line at 0.5 shows the median PFS, which indicates that at approximately 24 months, half of this group had experienced PrCa progression. But this was not seen in the non-persistent-tumor group. This stark difference in PrCa progression between the two subgroups is very significant, as indicated by the *p*-value less than 0.0001. This preliminary finding emphasizes the critical prognostic role of new tumor occurrence in predicting early progression and could inform closer surveillance or more aggressive follow-up therapies for patients who develop new tumors after initial treatment.

### 3.3. Statistical and Machine Learning Models for PFS

In this section, we present results from the optimized multivariate survival analysis models, namely PCM, RSF, and DeepSurv, along with their discriminatory ability as evaluated by their C-indices on both training and held-out test sets. Because variable selection was performed via regularization and model-specific importance criteria without post-selection inference adjustment, reported model estimates are interpreted as associated signals for risk stratification and not as causal or clinically actionable effect sizes. For PCM and RSF, reported discriminatory metrics represent the mean C-index across repeated model refits, with associated standard deviation reflecting between-run variability arising from internal resampling and hyperparameter tuning. Test set performance was evaluated on a fixed held-out dataset and was not resampled. For DeepSurv, model performance is reported from a single prescribed run with a fixed random seed and optimized hyperparameters selected within the training set; therefore, standard deviations are not applicable, and results are interpreted as complementary. C-indices are compared descriptively across models, as the study was not designed for formal hypothesis testing of model superiority. In each method, we only present the results from the final optimized model, while the full tuning procedures are provided in the Supplementary Materials (SF01_pipeline_modules_data; see the file multivariate-analysis.R).

#### 3.3.1. PCM

After model tuning was performed across a grid of elastic-net mixing parameter with (α ∈ [0,1]), using cross-validated Harrell’s C-Index within the training data. Moderate regularization (α = 0.5) achieved the highest cross-validated discrimination (C-Index = 0.86) while retaining six predictor variables with non-zero coefficients, yielding a predictive formulation for modeling PrCa PFS via the hazard function below:

(1) $$h\left(t\right)=h_{0}\left(t\right)\times exp\{2.28\times \;NTAIT+2.29\times HNT+1.41\times NCS+1.37\times MYH6\;+\;0.02\times WHS-1.07\times MSISS\}$$

Here, h(t) is the hazard function at time *t*, while $h_{0}(t)$ is the baseline hazard, and each coefficient represents the log hazard ratio (HR) for the respective covariate. The estimated hazard ratios of each selected predictor are shown in Table 3. These estimates are deemed as associative signals of relative risk stratification and should not be interpreted as causal or clinically actionable effect sizes.

Table 3 summarizes the coefficients retained under the optimized PCM. Reported HRs are provided to describe the direction and relative magnitude of associations within the training data rather than to support causal or clinically actionable interpretations. Several clinical variables exhibited elevated relative hazard estimates, including new tumor after initial treatment (NTAIT), history of neoadjuvant treatment (HNT), and neoplasm tumor cancer (NCS), indicating that patients with these characteristics were ranked at higher progression risk. Among the genomic features, the *MYH6* gene showed a positive hazard estimate, suggesting higher modeled progression risk with increasing tumor-normal deleterious SNV burden. As genomic predictors are defined on a sequence-derived scale, this association is interpreted as a relative risk contribution rather than a clinically standardized effect size. WHS demonstrated a modest positive association, while MSISS exhibited a negative hazard estimate, although the latter showed limited statistical strength. To assess the stability of discrimination performance, the PCM procedure was repeated across multiple resampling iterations within the training dataset while maintaining a fixed held-out test set. Across repeated model refits, average Harrell’s C-index values were 0.8442 on the training data and 0.8513 on the test data, indicating consistent risk-ranking performance on unseen data within the TCGA-PRAD cohort. Overall, the PCM procedure demonstrated that a small subset of clinical and genomic variables can provide relatively stable risk stratification for PFS with the TCGA-PRAD clinicogenomics cohort.

#### 3.3.2. RSF

RSF fitted on the clinicogenomics training dataset captured potential non-linear effects and interactions among predictors. Model hyperparameters were selected using internal out-of-bag (OOB) error minimization, yielding an optimal configuration with 20 trees, a terminal node size of seven, and eight variables randomly sampled at each split. Under this setting, RSF achieved a low OOB error (0.1181), indicating adequate internal risk ranking capability within the cohort. Across repeated model refits, average Harrell’s C-index values were 0.9080 on the training data and 0.8552 on the test data, indicating satisfactory risk-ranking within the TCGA-PRAD cohort.

The most influential variables selected by the RSF model based on the VIMP scores (see Table 4) include NTAIT, NCS, HNT, hypoxia-related scores (RHS and WHS) and mutation-burden clinical features (FGA and TMB), as well as the *MYH6, BRAF* and *TP53* tumor-normal SNV differences. Some clinical predictors identified by the RSF overlapped with those selected by the PCM, showing convergence across modeling approaches. Additional mutational and genomic features emerged uniquely in RSF; this is consistent with its capacity to capture latent or non-linear relationships. Variable influence measures are interpreted as relative contributions to model-based risk stratification rather than causal effects.

#### 3.3.3. DeepSurv

Using the optimized single-run DeepSurv configuration (one hidden layer with one node, dropout = 0.2, learning rate = 0.001, L2 = 0.2), the model achieved a training C-index of 0.8344 and a test C-Index of 0.8384, indicating satisfactory discriminatory ranking ability on an unseen held-out cohort [36,44]. For DeepSurv, we used gradient-based sensitivity scores to rank the importance of the clinicogenomics predictor variables, where higher scores indicate greater influence [42]. Table 5 below shows the sensitivity scores for all variables, both clinical and genomic variables contributed to model-based risk stratification with NCS, NTAIT, HNT and *MYH6* ranking consistent with findings from PCM and RSF models. However, gradient-based feature importance measures are model-dependent and do not represent causal effects; therefore, we interpret this conservatively, emphasizing features with consistent relevance across multiple modeling approaches. Given the modest sample size and event rate, we consider DeepSurv results as complementary and exploratory providers of additional perspective on clinicogenomics risk patterns rather than ultimate clinical prognostic conclusions.

### 3.4. Predicted Survival Probabilities and Risk Scores

In the context of PFS, survival probabilities for a time *t* represent the patients’ chances of cancer stabilization (i.e., no disease progression) for at least *t* months. Survival probabilities for PCM were obtained using the baseline survival function multiplied by the exponentiated linear risk score, while RSF survival probabilities are the exponential of the mean ensemble cumulative hazard function (CHF) across the ensemble of survival trees. Summary statistics for the predicted 6-year (72-month) survival probabilities for the patients in our test set are shown in Table 6. Our implementation of DeepSurv in R with Keras does not provide predicted survival probabilities but can calculate risk scores (see Table 6).

Risk scores across the models quantify each patient’s relative hazards for PrCa risk progression. In PCM, risk scores are calculated as the linear combinations of covariates weighted by the optimized regression coefficients, yielding log-relative hazard values and actual risk scores when exponentiated. For RSF, risk scores are derived as the cumulative hazard function aggregated over multiple survival trees, representing the expected risk over time. DeepSurv outputs a non-linear risk function trained via a neural network to approximate the Cox log-partial likelihood. In all cases, higher risk scores indicate greater susceptibility to disease progression or shorter duration of cancer stabilization. Summary statistics of all patient-level survival probabilities and risk scores are shown in Table 6, the upper rows for survival probabilities and the last 3 rows for risk scores.

Across all three modeling approaches, the median 6-year PFS (disease stabilization) was consistently high (median ≈ 0.90), indicating the majority of patients are expected to remain progression-free through the 6-year mark. However, the presence of very minimal survival probabilities (PCM: 0.00, RSF: 0.01) highlights a small but clinically important subgroup of patients at high-risk of early progression, underscoring substantial heterogeneity in patient trajectories.

Risk score distributions further clarified this heterogeneity. While all models produced positively skewed risk scores (mean > median), they identified higher-risk patients. DeepSurv-derived relative risk scores exhibited a median of 0.73 (IQR: 0.60–1.23), indicating that most patients were assigned lower than baseline progression risk, with a smaller subset displaying elevated risk. This moderately dispersed distribution suggests limited but meaningful risk stratification compared to PCM and RSF, which produced broader and more right-skewed distributions. DeepSurv demonstrated a more compressed risk range.

Accordingly, DeepSurv risk estimates are interpreted as complementary to the PCM and RSF models. While many patients exhibit indolent disease courses, those flagged as high-risk at 72 months across models may warrant closer monitoring or intensified treatment intervention. The overall concordance in survival probability estimates and the complementary nature of the risk stratification patterns across modeling approaches support the consistent findings within this cohort.

## 4. Discussion

This study investigated the potential prognostic utility of clinical and genomic features for predicting PFS in PrCa using three different modeling approaches: PCM, RSF and DeepSurv. In this section, we discuss the implications of our key findings by examining the contribution of genomics data to PFS prediction and the performance of the three models.

### 4.1. Contributions of Genomics Data to PFS Prediction

We integrated patient-level SNV information with clinical variables to construct a clinicogenomics dataset for PFS analysis. To assess the added value of genomic information, we applied the same modeling pipeline to both the clinicogenomics dataset and a clinical-only dataset, and summarized the variables identified as important by each modeling approach in Table 7. A consistent core set of clinical variables, HNT, NCS and NTAIT, which reflects neoadjuvant treatment history, neoplasm cancer status, and tumor recurrence, was repeatedly identified as influential for PFS risk stratification, regardless of whether genomic features were included. The convergence of these variables across PCM, RSF and DeepSurv models favors them as associative candidate markers of disease burden and risk intervention trajectory within this cohort. The incorporation of genomic variables enabled additional SNV-based signals to emerge, as the *MYH6* gene was also selected consistently across all models. Interestingly, *MYH6* is well known for its critical role in cardiac muscle contraction but had not been associated directly with PrCa until Wang et al. in 2024 reported that *MYH6* suppressed tumor progression in PrCa [45], which corroborated its importance in PFS prediction.

It should be emphasized that, at this stage, we focused only on interpreting features demonstrating cross-model relevance as associated signals influencing PrCa PFS risk stratification. Thus, clinicogenomics predictors that were not consistently selected across models are considered exploratory. Differences in predictor selection across models reflect expected methodological trade-offs, with linear penalized Cox models favoring parsimony and stability, and flexible machine learning models identifying a broader set of potential interacting features. Altogether, these results indicate that clinical variables remain the primary drivers of PFS risk stratification in this cohort, while genomic information provides complementary, hypothesis-generating insights.

### 4.2. Comparison of Model Performance

According to the C-indices shown in Table 8, all three models performed well on both training and test data. Any observed differences in C-index across models should be interpreted as indicative of relative discrimination within this cohort rather than a generalized statistically significant superiority. PCM showed consistent performance between training and test data, confirming favorable performance in ranking PFS risks. RSF performed well on the training set and slightly declined on the test set. The slight decline in C-index from training to test data is less than 0.1, suggesting acceptable for risk discrimination tasks [46,47]. Compared to PCM, RSF and DeepSurv demonstrated comparable test-set evaluation but showed greater sensitivity to model configuration. However, it should be noted that RSF and DeepSurv are complex models, which require a larger training dataset to achieve excellent performance. Table 8 also shows that the inclusion of genomic variables alongside clinical variables introduced greater complexity and, in some cases (e.g., DeepSurv), provided satisfactory risk stratification performance. Although the overall gains in C-Index were limited, the integration of genomic data provided complementary value without heavily compromising model performance.

Beyond individual model performance, the multi-model design of this study provides methodical insight into clinicogenomics survival modeling. Predictors that consistently emerge across models, particularly core clinical and select genomic variables, represent signals of PFS risk stratification, while model-specific findings may highlight areas where non-linear or higher-order interactions may exist. This approach demonstrates how heterogeneous modeling paradigms can be leveraged to balance interpretability with exploratory discovery in clinicogenomics analyses within this TCGA-PRAD cohort.

### 4.3. Multivariate Survival Models Versus Univariate KM Curves

**Figure 4:**
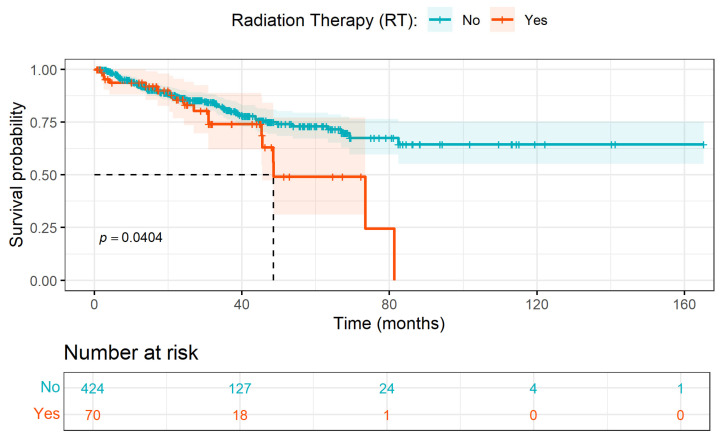
Kaplan–Meier survival curves showing progression-free survival (PFS) in months, stratified by radiation therapy status (Yes vs. No). Shaded regions indicate 95% confidence intervals, and tick marks denote censored observations. Dashed lines indicate the median PFS for the radiation therapy group. The number-at-risk table is shown below the main plot. Group differences were assessed using the log-rank test.

Univariate KM analyses were performed to provide descriptive summaries of unadjusted PFS patterns across selected clinical covariates. One counterintuitive pattern observed was the apparent association between radiation therapy (RT) and poorer PFS, with patients receiving RT exhibiting a steeper decline in unadjusted survival probabilities (see Figure 4), giving the impression that patients receiving RT experienced disease progression more rapidly (half had PrCa progressed at approximately 48 months) than those who did not. This result should not be interpreted as evidence of the detrimental effect of RT. Rather, it is most plausibly explained by confounding by indication [48], a common source of bias in observational studies where treatment assignment is not random. Patients receiving RT are more likely to have adverse disease characteristics, such as terminal tumor stage or greater clinical severity, which are not accounted for in univariate KM analyses. Thus, the KM curves presented are for the sole purpose of associative and explainable exploration and not to provide causal effects.

Consistent with this interpretation, RT was not retained as an important predictor in any of the multivariable survival models (PCM, RSF and DeepSurv) when evaluated alongside other clinical and genomic covariates. Thus, all inferential interpretations of covariate associations are restricted to multivariate survival modeling, with KM analyses serving a descriptive role only.

### 4.4. Limitations of the Study

This study is based on a single TCGA-PRAD cohort and therefore provides evidence of internally associated PFS risk stratification validity rather than external generalizability, while independent validation in external cohorts is needed prior to clinical translation. Since a fully nested cross-validation was not pursued, and also given the modest number of progression events, substantial right-censoring, and the use of machine learning modeling approaches, there is an inherent risk of overfitting despite the use of stratified train-test evaluation in DeepSurv and internal resampling in PCM and RSF. Thus, model performance evaluations are interpreted in terms of internally validated relative risk stratification rather than a generalized and precise prediction of progression events.

Clinical variables were imputed prior to data partitioning using a single completed dataset, and uncertainty due to imputation was not formally propagated, so imputed patterns should be deemed as exploratory. In addition, clinical and gene-level feature importance was assessed across multiple survival models without formal resampling or stability selection, and nonlinear models such as RSF and DeepSurv are sensitive to sample size and event rates; therefore, genomic findings and model-specific results are interpreted conservatively and will warrant confirmation in larger, externally validated datasets. Again, we state that gene-level importance was interpreted conservatively, with emphasis placed only on genomic features that exhibited consistent importance across all models.

## 5. Conclusions

We have explored the utility of combined clinical and genomic features in modeling PrCa PFS within a patient cohort using different statistical and machine learning models. The models consistently identified a core set of influential variables associated with PrCa progression, including the clinical variables HNT, NTAIT, and NCS, as well as the *MYH6* gene that is well known to be relevant to cardiac functions but only reported to be a tumor suppressor gene for PrCa progression relatively recently. These results suggest that integration of genomics with clinical data can help provide insights into PFS for patients with cancer.

It is noted that the modest cohort size, along with the lack of independent sets of genomics data and features for model assessment and validation, posed considerable limitations on the current study. However, the clinical and genomics variables consistently identified by multiple survival models to be associated with PrCa PFS can be useful for generating hypotheses for future experiments to uncover driving factors for cancer progression.

Future work will focus on extending validation in larger diverse cohorts with confounding analyses to help clarify counterintuitive treatment effects of RT, functional and biochemical pathway analyses of the *MYH6* gene in relation to PrCa progression, and the development of survival models to capture additional types of molecular data such as RNA and protein expression profiles from transcriptomics and proteomics data for the same TCGA cohort of patients with PrCa.

## Figures and Tables

**Table 1:** Clinical variables selected for analyses and their percentages of missing data.

Clinical Variable	Missing %
*PFS Status* ^a^	0.00
*PFS Months*	0.00
Age	0.00
History of Neoadjuvant Treatment (HNT)	0.00
International Classification of Diseases Histology (ICD-H)	0.00
MSI Mantis Score (MSIMS)	0.00
MSI Sensor Score (MSISS)	0.00
Prior Diagnosis (PD)	0.00
Tissue Source Site (TSS)	0.00
Tumor Mutation Burden (TMB)	0.00
Tumor Type (TT)	0.00
Mutation Count (MC)	0.61
Fraction of Genome Altered (FGA)	1.01
Pathological Tumor Stage (PTS)	1.42
Aneuploidy Score (AS)	4.66
Radiation Therapy (RT)	9.31
New Tumor After Initial Treatment (NTAIT)	11.74
Pathological Node Stage (PNS)	14.78
Neoplasm Cancer Status (NCS)	17.41
Buffa Hypoxia Score (BHS)	32.59
Ragnum Hypoxia Score (RHS)	32.59
Winter Hypoxia Score (WHS)	32.59

^a^ The target variables of interest *PFS Status* and *PFS Month* for PFS modelling are italicized.

**Table 2:** Summary statistics of PFS time in censored and non-censored groups.

PFS Status	N	Mean	SD	Minimum	Q1	Median	Q3	Maximum
Censored	401	33.5	25.8	0.76	15.6	27.8	45.5	165.2
Progression	93	23.8	19.0	1.68	8.71	18.4	34.8	82.4

**Table 3:** Optimized PCM summary: SE = standard error. Hazard ratios are reported for descriptive and associative signals only. Due to the penalized variable selection, sequencing-derived feature scales, and lack of post selection inference adjustment, these estimates should not be interpreted as causal or clinically actionable effect sizes.

Variable	Coefficient (SE)	HR	*p*-Value
NTAIT	2.28 (0.33)	9.78	*p* < 0.001
HNT	2.28 (0.78)	9.78	*p* < 0.01
NCS	1.41 (0.35)	4.10	*p* < 0.001
*MYH6*	1.37 (0.46)	3.94	*p* < 0.01
WHS	0.02 (0.01)	1.02	*p* < 0.05
MSISS	−1.07 (0.56)	0.34	*p* < 0.1

**Table 4:** Important covariates identified by RSF model and their VIMP scores.

Covariates	VIMP Scores
NTAIT	0.50
NCS	0.13
WHS	0.13
*BRAF* Gene	0.07
HNT	0.09
FGA	0.06
TMB	0.05
RHS	0.04
*MYH6* Gene	0.04
*TP53* Gene	0.02

**Table 5:** Important clinicogenomics features ranked by gradient-based feature attribution scores.

Covariates	Importance Score
NCS	0.6773
NTAIT	0.3463
HNT	0.3134
PTS	0.2750
ICD-H	0.2010
*MYH6* gene	0.1920
WHS	0.1242
FGA	0.1224
PNS	0.1208
*KMT2C* gene	0.1139
*CTNNB1* gene	0.1128
TSS	0.1049
TT	0.0832
RHS	0.0780
*LRP1B* gene	0.0772

**Table 6:** Summary of patient-level survival probabilities and risk scores.

Survival Probabilities and Risk Scores	Mean	SD	Min	Q1	Med	Q3	Max
6-year PCM survival probability	0.74	0.31	0.00	0.68	0.90	0.92	0.98
6-year RSF survival probability	0.74	0.33	0.01	0.59	0.91	0.98	1.00
PCM Risk scores	7.86	17.0	0.19	0.88	1.14	4.13	123
RSF Risk scores	9.38	15.60	0.00	0.30	1.62	7.80	57.2
DeepSurv risk scores	0.95	0.51	0.47	0.60	0.73	1.23	2.84

**Table 7:** Shortlisted important predictors for PrCa PFS using clinicogenomics data in comparison to only clinical data across all models, displaying associative influential signals identified as important for PFS across PCM, RSF and DeepSurv models using clinicogenomics and clinical only datasets. A filled dot (●) indicates that a predictor was selected as influential by the corresponding model under its optimized configuration. Predictor selection reflects model-specific importance criteria (non-zero coefficients for PCM, VIMP, and RSF, and gradient-based sensitivity scores for DeepSurv). Variables highlighted in green across multiple models are interpreted as more influential associative signals (HNT, NCS, NTAIT, and *MYH6* gene), whereas predictors not consistently selected across models are considered exploratory. The area under Clinical Data Only columns for the genomics predictors at the bottom are shaded in grey as these predictors are not present in the clinical only dataset.

Predictor	Clinicogenomics Data			Clinical Data Only		
	PCM	RSF	DeepSurv	PCM	RSF	DeepSurv
HNT	●	●	●	●	●	●
NCS	●	●	●	●	●	●
NTAIT	●	●	●	●	●	●
WHS	●	●	●	●	●	
FGA		●	●			●
RHS		●	●		●	
MSISS	●				●	●
PTS			●	●		●
ICD-H			●			●
TT			●			●
PNS			●			
TMB		●				
TSS			●			
Age						●
MC				●		
RT						●
*MYH6*	●	●	●			
*BRAF*		●				
*CTNNB1*			●			
*KMT2C*			●			
*LRP1B*			●			
*TP53*		●				

**Table 8:** C-Indices across different models for clinicogenomics and clinical datasets, reporting the mean C-index and standard deviation (SD) across repeated model refits and internal resampling within the training set, with performances evaluated on a fixed held-out test set. SD for the test set reflects between-run variability rather than uncertainty from test set resampling. DeepSurv performance is reported from a single prespecified training run with fixed random seed and hyperparameters selected via cross-validation within the training set. Given the stochastic nature of neural network optimization and the modest sample size, we deem DeepSurv results as complementary.

Model	Clinicogenomics		Clinical Only	
	Training [SD]	Testing [SD]	Training [SD]	Testing [SD]
PCM	0.84 [0.01]	0.85 [0.00]	0.86 [0.01]	0.85 [0.00]
RSF	0.90 [0.02]	0.86 [0.01]	0.93 [0.01]	0.86 [0.01]
DeepSurv	0.83	0.84	0.84	0.81

## Data Availability

The clinical data used in this work were freely obtained from the TCGA Prostate Adenocarcinoma (PRAD) Pan-Cancer Atlas (2018) study, through the cBioPortal for Cancer Genomics at https://www.cbioportal.org/study/clinicalData?id=prad_tcga_pan_can_atlas_2018 (accessed on 11 October 2022). SNV data of the patient cohort can be accessed via the NIH GDC portal https://portal.gdc.cancer.gov/projects/TCGA-PRAD (accessed on 11 October 2022). The compiled clinicogenomics data for this study is available at the GitHub repository: https://github.com/kelvin-meyet/ClinicoGenomicInsights/blob/main/SF01_pipeline_modules_data/prca_clinicogenomics_data.csv (accessed on 16 December 2025).

## Supplementary Materials

The following supporting information can be downloaded at: https://www.mdpi.com/article/10.3390/ijerph23020256/s1. Supplementary Materials (SF01_pipeline_modules_data, SF02_data_processing, SF03_clinical_variables, SF04_imputation, SF05_kmcurves_and_plots) can be downloaded from the project’s GitHub repository: https://github.com/kelvin-meyet/ClinicoGenomicInsights (accessed on 9 September 2025).

## Abbreviations

The following abbreviations were used in this manuscript:

PrCa	Prostate Cancer
PFS	Progression Free Survival
OS	Overall Survival
SNVs	Single Nucleotide Variants
PCM	Penalized Cox Model
RSF	Random Survival Forest
DeepSurv	Deep Survival Neural Network
SF	Supplementary File
REST API	Representative State Transfer Application Programming Interface
TCGA-PRAD	The Cancer Genome Atlas Prostate Adenocarcinoma
MICE	Multivariate Imputation by Chained Equations
PMM	Predictive Mean Matching
RF	Random Forest
VCF	Variant Call Format
FATHMM	Functional Analysis through Hidden Markov Models
PROVEAN	Protein Variation Effect Analyzer
GO	Gene Ontology
KEGG	Kyoto Encyclopedia of Genes and Genome
LPC gene	Likely PrCa Gene
ID	Identifiers
KM	Kaplan–Meier
C-Index	Concordance Index
CV	Cross Validation
SELU	Scaled Exponential Linear Unit
NTAIT	New Tumor after Initial Treatment
RT	Radiation Therapy
HNT	History of Neoadjuvant Treatment
MSISS	Mutation Satellite Instability Sensor Score
TMB	Tumor Mutation Burden
NCS	Neoplasm Cancer Status
RHS	Ragnum Hypoxia Score
WHS	Winter Hypoxia Score
SE	Standard Error
HR	Hazard Ratio
VIMP	Variable Importance
CHF	Cumulative Hazard Function
RNA	Ribonucleic Acid

## References

[1] Mayo Clinic (2025). Metastatic Prostate Cancer—Symptoms and Causes. https://www.mayoclinic.org/diseases-conditions/metastatic-prostate-cancer/symptoms-causes/syc-20377966.

[2] Healthline (2023). Aggressive Prostate Cancer: What It Is and How It’s Treated. https://www.healthline.com/health/prostate-cancer/aggressive-prostate-cancer.

[3] American Cancer Society (2025). Cancer Facts & Figures 2025.

[4] Siegel R.L., Kratzer T.B., Giaquinto A.N., Sung H., Jemal A. (2025). Cancer statistics, 2025. CA A Cancer J. Clin..

[5] Becerra M.F., Atluri V.S., Bhattu A.S., Punnen S. (2020). Serum and urine biomarkers for detecting clinically significant prostate cancer. Urol. Oncol. Semin. Orig. Investig..

[6] Vlajnic T., Bubendorf L. (2020). Molecular pathology of prostate cancer: A practical approach. Pathology.

[7] Corres-Mendizabal J., Zacchi F., Martín-Martín N., Mateo J., Carracedo A. (2024). Metastatic hormone-naïve prostate cancer: A distinct biological entity. Trends Cancer.

[8] Yamaguchi T.N., Houlahan K.E., Zhu H., Kurganovs N., Livingstone J., Fox N.S., Yuan J., Sietsma Penington J., Jung C.-H., Schwarz T. (2025). The Germline and Somatic Origins of Prostate Cancer Heterogeneity. Cancer Discov..

[9] Leslie S.W., Soon-Sutton T.L., Skelton W.P. (2025). Prostate Cancer. StatPearls.

[10] Halabi S., Roy A., Rydzewska L., Guo S., Godolphin P., Hussain M., Tangen C., Thompson I., Xie W., Carducci M.A. (2024). Radiographic Progression-Free Survival and Clinical Progression-Free Survival as Potential Surrogates for Overall Survival in Men with Metastatic Hormone-Sensitive Prostate Cancer. J. Clin. Oncol..

[11] Belin L., Tan A., De Rycke Y., Dechartres A. (2020). Progression-free survival as a surrogate for overall survival in oncology trials: A methodological systematic review. Br. J. Cancer.

[12] Hatano K., Nonomura N. (2022). Genomic Profiling of Prostate Cancer: An Updated Review. World J. Men’s Health.

[13] Chen R., Tang L., Melendy T., Yang L., Goodison S., Sun Y. (2024). Prostate Cancer Progression Modeling Provides Insight into Dynamic Molecular Changes Associated with Progressive Disease States. Cancer Res. Commun..

[14] Das R., Sjöström M., Shrestha R., Yogodzinski C., Egusa E.A., Chesner L.N., Chen W.S., Chou J., Dang D.K., Swinderman J.T. (2021). An integrated functional and clinical genomics approach reveals genes driving aggressive metastatic prostate cancer. Nat. Commun..

[15] Ozay Z.I., Agarwal N. (2025). Race, Ethnicity, and Tumor Genomic Testing in Prostate Cancer. JAMA Netw. Open.

[16] He Y., Zhang J., Chen Z., Sun K., Wu X., Wu J., Sheng L. (2022). A seven-gene prognosis model to predict biochemical recurrence for prostate cancer based on the TCGA database. Front. Surg..

[17] Pellegrini M. (2023). Accurate prognosis for localized prostate cancer through coherent voting networks with multi-omic and clinical data. Sci Rep..

[18] Cerami E., Gao J., Dogrusoz U., Gross B.E., Sumer S.O., Aksoy B.A., Jacobsen A., Byrne C.J., Heuer M.L., Larsson E. (2012). The cBio cancer genomics portal: An open platform for exploring multidimensional cancer genomics data. Cancer Discov..

[19] Gao J., Aksoy B.A., Dogrusoz U., Dresdner G., Gross B., Sumer S.O., Sun Y., Jacobsen A., Sinha R., Larsson E. (2013). Integrative analysis of complex cancer genomics and clinical profiles using the cBioPortal. Sci. Signal..

[20] van Buuren S., Groothuis-Oudshoorn K. (2011). mice: Multivariate Imputation by Chained Equations in R. J. Stat. Softw..

[21] Wang B., Mohl J., Leung M.-Y. Computational Prediction of Functional Effects for Cancer Related Genetic Sequence Variants. Proceedings of the 2020 IEEE International Conference on Bioinformatics and Biomedicine (BIBM).

[22] Rogers M.F., Shihab H.A., Mort M., Cooper D.N., Gaunt T.R., Campbell C. (2018). FATHMM-XF: Accurate prediction of pathogenic point mutations via extended features. Bioinformatics.

[23] Choi Y., Chan A.P. (2015). PROVEAN web server: A tool to predict the functional effect of amino acid substitutions and indels. Bioinformatics.

[24] Wang B. (2021). Identification Of Prostate Cancer-Associated Genomic Alterations By Analyzing Variant Frequencies, Functional Effects, And Protein Interactions. Doctoral Dissertation.

[25] Kaplan E.L., Meier P., Kotz S., Johnson N.L. (1992). Nonparametric Estimation from Incomplete Observations. Breakthroughs in Statistics: Methodology and Distribution.

[26] Peto R., Peto J. (1972). Asymptotically Efficient Rank Invariant Test Procedures. J. R. Stat. Society. Ser. A (Gen.).

[27] Harrell F.E., Califf R.M., Pryor D.B., Lee K.L., Rosati R.A. (1982). Evaluating the yield of medical tests. JAMA.

[28] Schmid M., Wright M.N., Ziegler A. (2016). On the use of Harrell’s C for clinical risk prediction via random survival forests. Expert Syst. Appl..

[29] Park S.Y., Park J.E., Kim H., Park S.H. (2021). Review of Statistical Methods for Evaluating the Performance of Survival or Other Time-to-Event Prediction Models (from Conventional to Deep Learning Approaches). Korean J. Radiol..

[30] Tay J.K., Narasimhan B., Hastie T. (2023). Elastic Net Regularization Paths for All Generalized Linear Models. J. Stat. Softw..

[31] Spytek M., Krzyziński M., Langbein S.H., Baniecki H., Wright M.N., Biecek P. (2023). survex: An R package for explaining machine learning survival models. Bioinformatics.

[32] Ishwaran H., Kogalur U.B., Blackstone E.H., Lauer M.S. (2008). Random survival forests. Ann. Appl. Stat..

[33] Ishwaran H., Kogalur U.B. (2023). Fast Unified Random Forests for Survival, Regression, and Classification (RF-SRC). Manual. https://cran.r-project.org/package=randomForestSRC.

[34] Ishwaran H., Lu M., Kogalur U.B. (2021). RandomForestSRC: Variable Importance (VIMP) with Subsampling Inference Vignette. RFSRC.

[35] Ishwaran H., Chen X., Minn A.J., Lu M., Lauer M.S., Kogalur U.B. (2021). RandomForestSRC: Minimal Depth Vignette. RFSRC.

[36] Katzman J.L., Shaham U., Cloninger A., Bates J., Jiang T., Kluger Y. (2018). DeepSurv: Personalized treatment recommender system using a Cox proportional hazards deep neural network. BMC Med. Res. Methodol..

[37] Li J. (2024). Jinli-Stat/DeepSurv-R-Keras. https://github.com/jinli-stat/DeepSurv-R-Keras.

[38] Kingma D.P., Ba L.J. (2015). Adam: A Method for Stochastic Optimization. https://dare.uva.nl/search?identifier=a20791d3-1aff-464a-8544-268383c33a75.

[39] Klambauer G., Unterthiner T., Mayr A., Hochreiter S. (2017). Self-Normalizing Neural Networks. arXiv.

[40] Nesterov Y. (2013). Gradient methods for minimizing composite functions. Math. Program..

[41] Senior A., Heigold G., Ranzato M., Yang K. (2013). An empirical study of learning rates in deep neural networks for speech recognition. Proceedings of the 2013 IEEE International Conference on Acoustics, Speech and Signal Processing (ICASSP).

[42] Simonyan K., Vedaldi A., Zisserman A. (2013). Deep Inside Convolutional Networks: Visualising Image Classification Models and Saliency Maps. arXiv.

[43] Sundararajan M., Taly A., Yan Q. Axiomatic Attribution for Deep Networks. Proceedings of the International Conference on Machine Learning.

[44] López O.A.M., López A.M., Crossa D.J. (2022). Overfitting, Model Tuning, and Evaluation of Prediction Performance. Multivariate Statistical Machine Learning Methods for Genomic Prediction.

[45] Wang F., Shen H., Li K., Ding Y., Wang J., Sun J. (2024). MYH6 suppresses tumor progression by downregulating KIT expression in human prostate cancer. Sci. Rep..

[46] Goodfellow I., Bengio Y., Courville A. (2016). Deep Learning.

[47] Hastie T., Tibshirani R., Friedman J.H. (2009). The Elements of Statistical Learning: Data Mining, Inference, and Prediction.

[48] Kyriacou D.N., Lewis R.J. (2016). Confounding by Indication in Clinical Research. JAMA.

