# Supplementary material for: Clinicogenomic Insights for Progression-Free Survival in Prostate Cancer
Source: Int J Environ Res Public Health. 2026 Feb 18;23(2):256. doi: 10.3390/ijerph23020256 (PMC12940860; doi:10.3390/ijerph23020256)
Supplement: Supplementary file 1 [file ijerph-23-00256-s001.zip › SF03_clinical_variables.pdf]

Clinical Variable	Type of Variable	Definition
**Age**	Numeric	Age of patient in years
**Buffa Hypoxia Score**	Numeric	Gene expression-based signatures developed to quantify oxygen content in a tumor. (Buffa et al., 2010).
**History of Neoadjuvant Treatment**	Categorical<br>(No = 1; Yes = 2)	Treatment given for cancer before the main treatment.(*NCI Dictionary of Cancer Terms*, 2011).
**ICD Histology**	Categorical<br>(Acina cell carcinoma = 1; adenocarcinoma = 2; infiltrate_duel_carc = 3; mixed adenocarcinoma = 4; mucinous adenocarcinoma = 5; signet ring cell carc. = 6)	Codes to identify site (topography) and the histology (morphology) of neoplasms. (Allen, 1991).
**Prior Diagnosis**	Categorical<br>(1 = No; 2 = Yes)	Patients with a prior diagnosis
**New Tumor After Initial Diagnosis**	Categorical<br>(1 = No; 2 = Yes)	Persistence of tumor after initial treatment.
**Pathological Node Stage**	Categorical<br>(1 = cancer in lymph nodes<br>0 = cancer not in lymph nodes)	Cancer spread to lymph nodes.<br>(*Cancer Staging - NCI*, 2015)
**Pathological Tumor Stage**	Categorical<br>(T2A = Tumor is between 2-5 cm across and in either half or less of the prostate.<br>T2B = Tumor is between 2-5 cm across and in either half or more of the prostate<br>T2C = Tumor is between 2-5 cm across and in both sides of prostate<br>T3A = Tumor is more than 5cm across in either half or less of the prostate.<br>T4 = Tumor is of other size and spreading into other tissues)	Size of the cancer and how far it has spread into nearby tissue.<br>(Canadian Cancer Society, 2021; *Cancer Staging - NCI*, 2015)
**Neoplasm Cancer Status**	Categorical<br>(1 = Tumor free; 2 = With tumor)	Status of abnormal tumor growth.<br>(*NCI Dictionary of Cancer Terms*, 2011)
**Radiation Therapy**	Categorical<br>(1 = No; 2 = Yes)	Individuals exposed to radiation therapy treatment
**Ragnum Hypoxia Score**	Numeric	Prostate cancer specific hypoxia gene signatures developed to assess tumor oxygen levels.<br>(Ragnum et al., 2015; Yang et al., 2018)

**Winter Hypoxia Score**	Numeric	Gene expression signatures developed to quantify tumor oxygen levels across cancer types. (Winter et al., 2007)
**Aneuploidy Score**	Numeric	Measure the degree of aneuploidy (abnormal number of chromosomes) in a cell or tumor (A. M. Taylor et al., 2018).
**MSI Sensor Score**	Numeric	Determines the genomic instability by gauging the level of microsatellite instability of tumors. (Niu et al., 2014).
**MSI mantis score**	Numeric	Method for predicting microsatellite instability (MSI) status in tumor cells.<br>(Ye et al., 2023)
**Tissue Source Site**	Categorical<br>(1 = Biotech & Pharmaceutical company<br>2 = Hospital<br>3 = Research Centre<br>4 = University	Site where blood, tissues and patient metadata are obtained (Cerami et al., 2012).
**Tumor Type**	Categorical<br>(1 = Prostate adenocarcinoma acinar<br>2 = Prostate adenocarcinoma other subtype)	The morphologic changes of prostate carcinoma variants.<br>(Cerami et al., 2012; Li & Wang, 2016)
**Tumor Mutation Burden**	Numeric	Total number of somatic non-synonymous mutations present within the cancer genome. (Budczies et al., 2024).
**Fraction of Genome Altered**	Numeric	Proportion of tumor genome altered by genetic mutation. (Cerami et al., 2012; Plantalech, 2018)
**Mutation Count**	Numeric	Count of mutations found in the tumor genome. (Cerami et al., 2012; Plantalech, 2018)
**PFS Months**	Numeric	Duration (months) where cancer ceases progression. (Gyawali et al., 2022; Lebwohl et al., 2009).
**PFS Status**	Categorical<br>(0 = Censored; 1 = Progression)	Indicators to determine whether cancer progressed (1) or no information about cancer status (0).
