# Supplementary material for: Clinicogenomic Insights for Progression-Free Survival in Prostate Cancer
Source: Int J Environ Res Public Health. 2026 Feb 18;23(2):256. doi: 10.3390/ijerph23020256 (PMC12940860; doi:10.3390/ijerph23020256)
Supplement: Supplementary file 1 [file ijerph-23-00256-s001.zip › SF04_imputation.pdf]

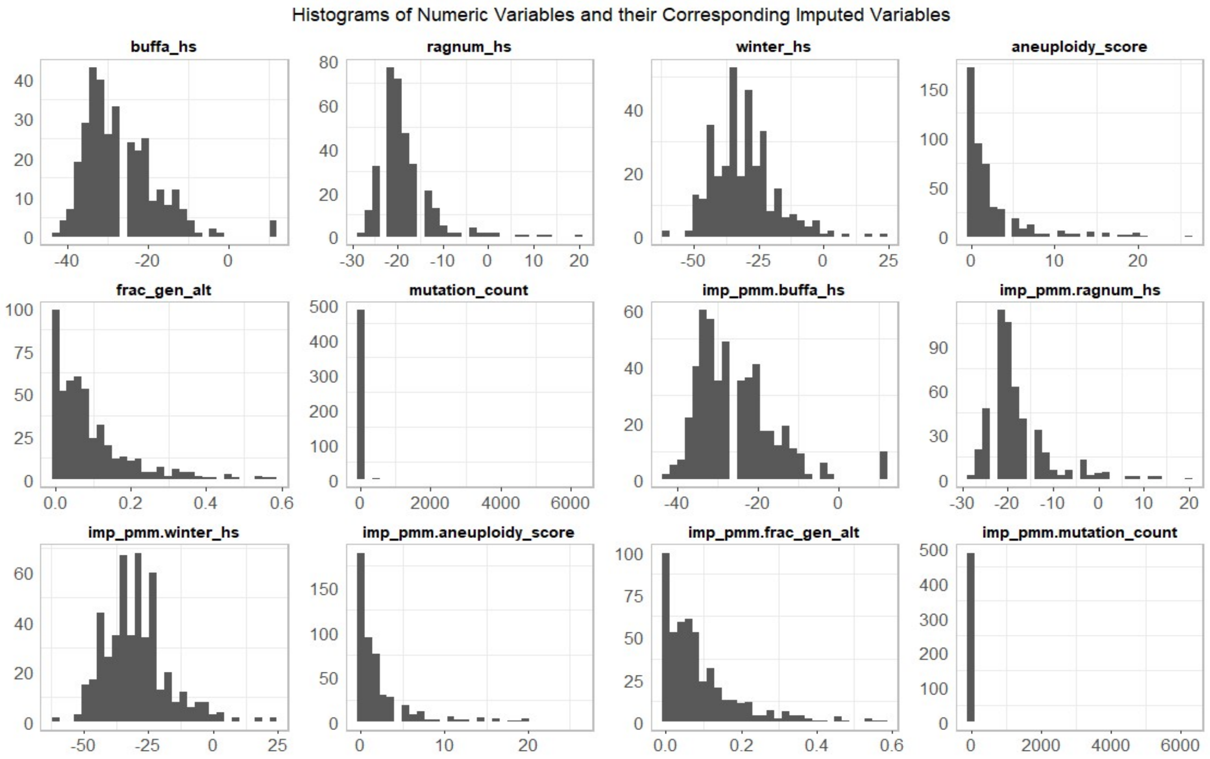
Histograms of Numeric Variables and their Corresponding Imputed Variables
buffa_hs
ragnum_hs
winter_hs
aneuploidy_score
frac_gen_alt
mutation_count
imp_pmm.buffa_hs
imp_pmm.ragnum_hs
imp_pmm.winter_hs
imp_pmm.aneuploidy_score
imp_pmm.frac_gen_alt
imp_pmm.mutation_count

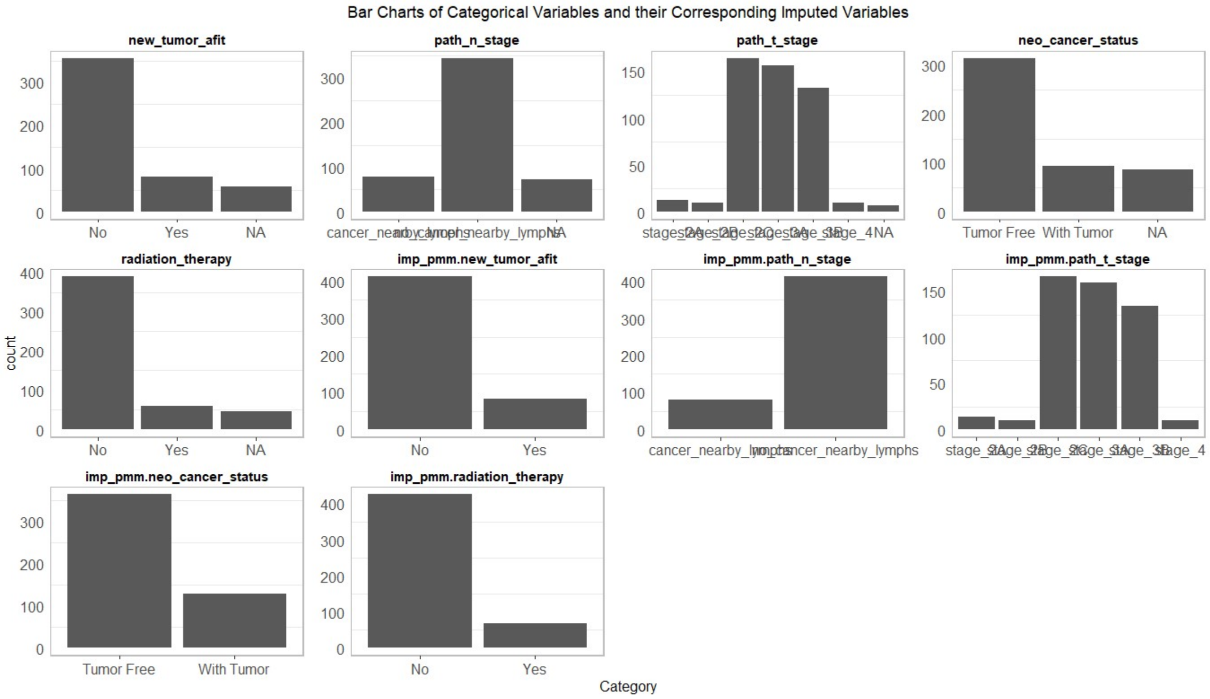
Bar Charts of Categorical Variables and their Corresponding Imputed Variables
new_tumor_afit
path_n_stage
path_t_stage
neo_cancer_status
radiation_therapy
imp_pmm.new_tumor_afit
imp_pmm.path_n_stage
imp_pmm.path_t_stage
imp_pmm.neo_cancer_status
imp_pmm.radiation_therapy
No
Yes
NA
Tumor Free
With Tumor
count
Category

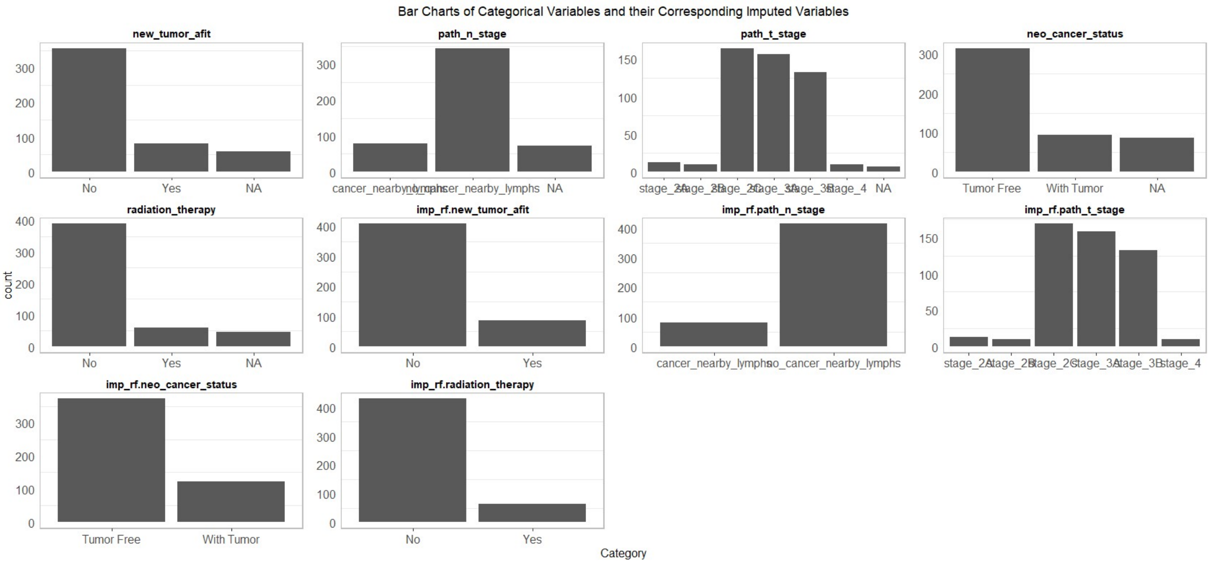
Bar Charts of Categorical Variables and their Corresponding Imputed Variables
new_tumor_afit
path_n_stage
path_t_stage
neo_cancer_status
radiation_therapy
imp_rf.new_tumor_afit
imp_rf.path_n_stage
imp_rf.path_t_stage
imp_rf.neo_cancer_status
imp_rf.radiation_therapy
count
Category
No
Yes
NA
cancer_nearby_lymphs
no_cancer_nearby_lymphs
stage_4
Tumor Free
With Tumor

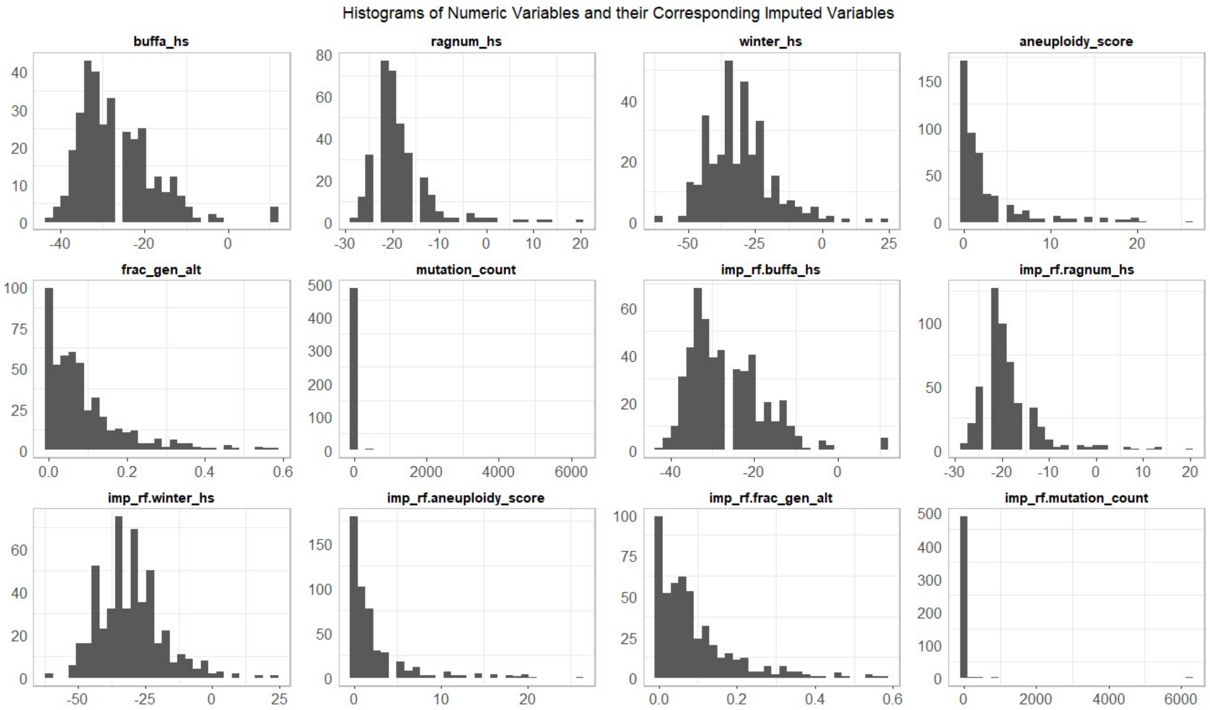
Histograms of Numeric Variables and their Corresponding Imputed Variables
buffa_hs
ragnum_hs
winter_hs
aneuploidy_score
frac_gen_alt
mutation_count
imp_rf.buffa_hs
imp_rf.ragnum_hs
imp_rf.winter_hs
imp_rf.aneuploidy_score
imp_rf.frac_gen_alt
imp_rf.mutation_count

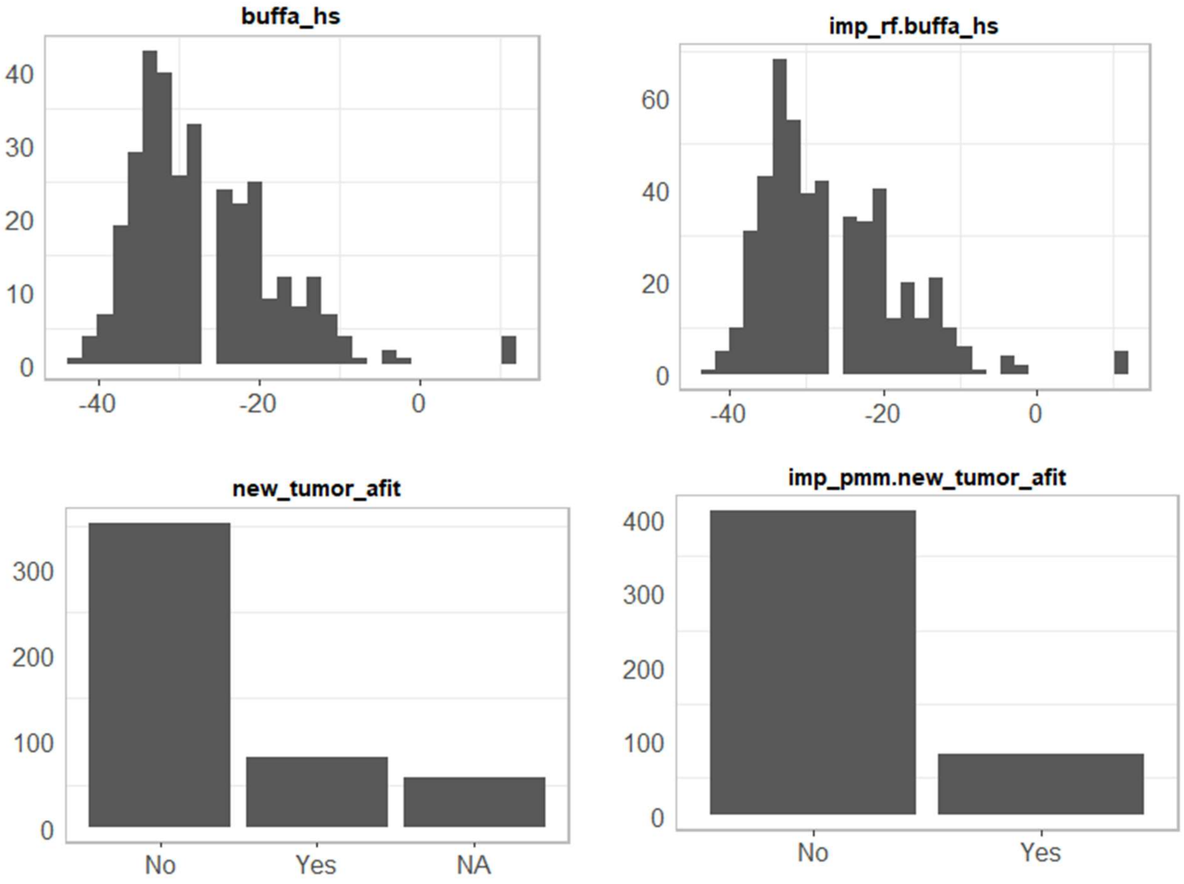

buffa_hs
imp_rf.buffa_hs
new_tumor_afit
imp_pmm.new_tumor_afit
No
Yes
NA
