# Supplementary material for: Clinicogenomic Insights for Progression-Free Survival in Prostate Cancer
Source: Int J Environ Res Public Health. 2026 Feb 18;23(2):256. doi: 10.3390/ijerph23020256 (PMC12940860; doi:10.3390/ijerph23020256)
Supplement: Supplementary file 1 [file ijerph-23-00256-s001.zip › SF05_kmcurves_and_plots.pdf]

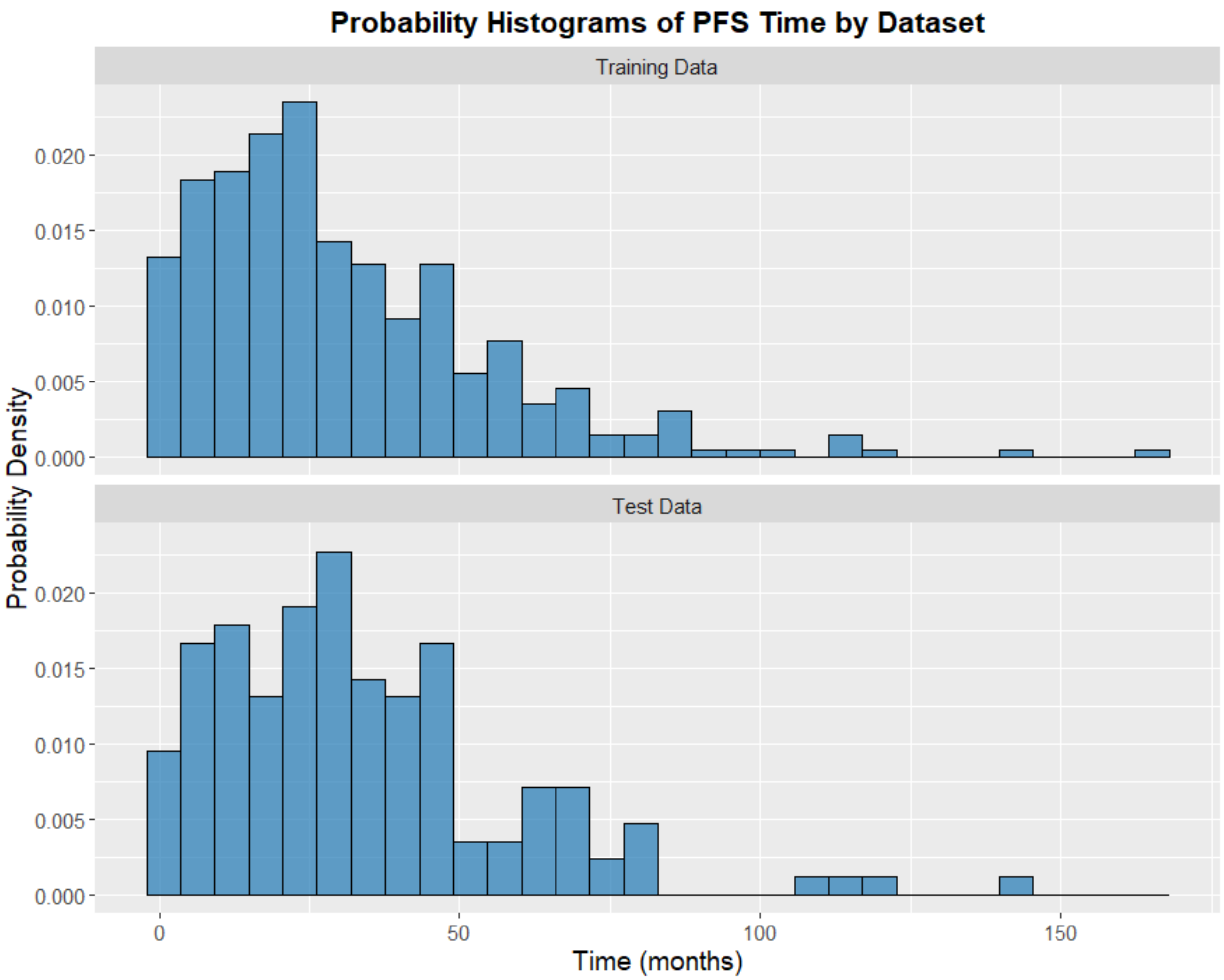

Figure S1. Probability density histograms of progression-free survival (PFS) time for the training and test dataset

Figure S1. Both distributions exhibit similar shape and spread, indicating that the test cohort is representative of the training set. This minimizes sampling bias and supports the generalizability of predictive survival modeling.

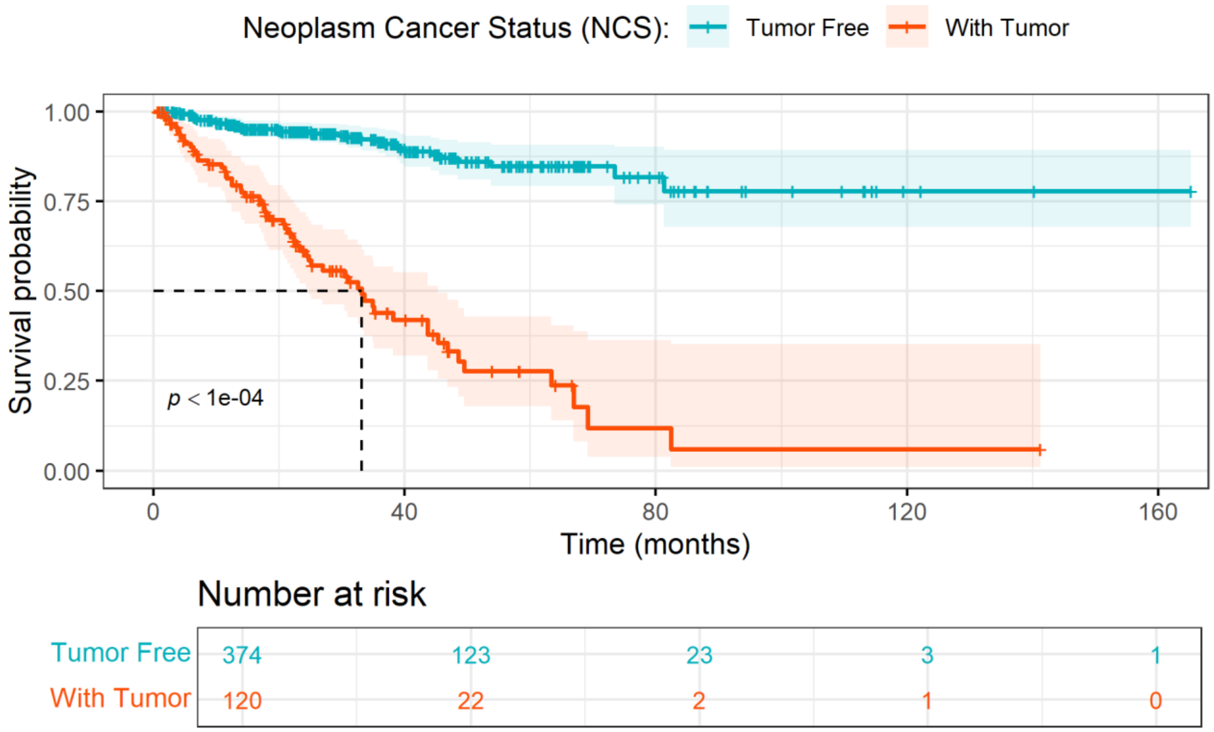

Figure S2. Kaplan-Meier survival curve showing progression-free survival by neoplasm cancer status with logrank test.

Figure B illustrates PFS stratified by neoplasm cancer status (comparing patients who are tumor free versus those with tumor). The tumor-free group shows a relatively high and stable progression free survival over time for longer periods, their PFS stabilizes above 75% indicating sustained disease stabilization. The tumor group experiences a sharp decline in PFS particularly within the first 40 months. PFS falls below the median survival at early stages and continues to fall overtime, indicating early and frequent chances of disease progression with increased uncertainty as confidence limits get wider over time. This demonstrates that patients who are tumor-free exhibit significantly higher PrCa PFS compared to those with the tumor in this cohort.

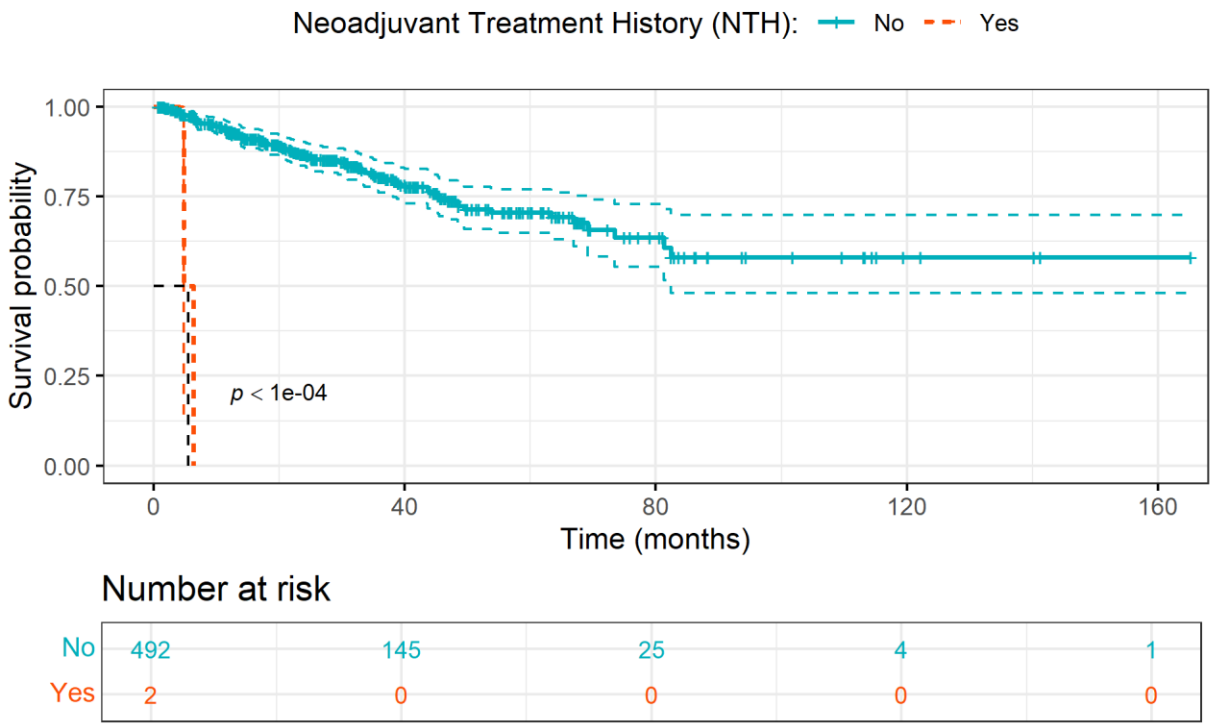

Figure S3. Kaplan-Meier survival curve showing progression-free survival by neoadjuvant treatment history

This KM curve in figure C suggests that patients who did not have history neoadjuvant treatment exhibited significantly longer PFS compared to those with historical neoadjuvant treatment. This is seen by the early and total drop in PFS probability among patients who had neoadjuvant treatment history. However, the extreme limited sample size (n=2) for the "Yes" group (historical neoadjuvant treatment) severely cast doubts on the plausibility of this preliminary finding and raises suspicions of lack of representativeness for this subgroup.
